# When Immunophenotype Is Not Identity: A Clinicopathological Review of Neuroendocrine Differentiation in Tumors of the Female Genital Tract

**DOI:** 10.3390/diagnostics16101573

**Published:** 2026-05-21

**Authors:** Catalin-Bogdan Satala, Alina-Mihaela Gurau, Gabriela Patrichi, Roxana-Cristina Mehedinti, Andy Radu Leibovici, Gabriela Gurau

**Affiliations:** 1 Faculty of Medicine and Pharmacy, Medical and Pharmaceutical Research Center, “Dunarea de Jos” University of Galati, 800008 Galati, Romania; catalin.satala@ugal.ro (C.-B.S.); roxana.mehedinti@ugal.ro (R.-C.M.); andy.leibovici@ugal.ro (A.R.L.); gabriela.gurau@ugal.ro (G.G.); 2 Department of Pathology, Clinical County Emergency Hospital Braila, 810325 Braila, Romania; 3 The School for Doctoral Studies in Biomedical Sciences, “Dunarea de Jos” University of Galati, 800008 Galati, Romania; 4 The Doctoral School of Medicine and Pharmacy, “George Emil Palade” University of Medicine, Pharmacy, Science and Technology, 540142 Targu Mures, Romania; 5 “Sf. Ioan” Clinical Emergency Pediatric Hospital, 800487 Galati, Romania

**Keywords:** neuroendocrine differentiation, female genital tract tumors, gynecologic pathology, neuroendocrine carcinoma, immunophenotype, immunohistochemistry, diagnostic pitfalls, mixed neuroendocrine/non-neuroendocrine tumors

## Abstract

Neuroendocrine differentiation in tumors of the female genital tract is an uncommon but diagnostically consequential finding. Its interpretation is challenging because neuroendocrine marker expression does not necessarily define a neuroendocrine neoplasm. Focal or aberrant staining for synaptophysin, chromogranin A, CD56 or INSM1 may occur in otherwise conventional gynecologic carcinomas, whereas true poorly differentiated neuroendocrine carcinomas represent aggressive tumors with distinct prognostic and therapeutic implications. This narrative review examines neuroendocrine differentiation across the cervix, endometrium, ovary, vagina and vulva from an integrated clinicopathologic perspective. We emphasize that neuroendocrine differentiation should be approached as a diagnostic and biological spectrum, ranging from incidental immunophenotypic expression to carcinoma with neuroendocrine differentiation, mixed neuroendocrine/non-neuroendocrine tumors, well-differentiated neuroendocrine tumors and poorly differentiated neuroendocrine carcinomas. Morphology remains the diagnostic anchor, while immunohistochemistry, molecular context and clinicoradiologic correlation refine classification and help exclude mimics or metastatic disease. Site-specific interpretation is essential: cervical neuroendocrine carcinoma is commonly HPV-associated and clinically aggressive; endometrial tumors require integration with p53, mismatch repair, *POLE* and SWI/SNF-related contexts; ovarian lesions demand distinction between primary well-differentiated neuroendocrine tumors, poorly differentiated carcinomas and metastases; and vaginal or vulvar tumors require careful exclusion of adjacent extension, cutaneous mimics and extragenital primaries. We propose a practical diagnostic framework that separates incidental marker expression from clinically meaningful neuroendocrine differentiation and links this distinction to reporting, prognosis and treatment. The central diagnostic question is not whether neuroendocrine markers are expressed but whether their expression defines a morphologically, biologically and clinically meaningful tumor category.

## 1. Introduction

Neuroendocrine differentiation in tumors of the female genital tract represents an uncommon but clinically relevant phenomenon, ranging from focal neuroendocrine marker expression in otherwise conventional carcinomas to bona fide neuroendocrine neoplasms with distinct biological and clinical behavior [1,2]. Although neuroendocrine tumors and neuroendocrine carcinomas are well recognized in the lung, gastrointestinal tract and pancreas, their occurrence in gynecologic sites is rare and remains less well characterized. Neuroendocrine differentiation has been described in tumors of the cervix, endometrium, ovary, vagina and vulva, but its significance varies considerably according to tumor type, anatomic site and clinicopathologic context [3,4].

Poorly differentiated neuroendocrine carcinomas of the female genital tract are generally aggressive neoplasms, characterized by high-grade morphology, brisk mitotic activity, necrosis, early lymphovascular invasion and a propensity for nodal and distant dissemination [2,4]. Cervical neuroendocrine carcinoma, particularly small-cell neuroendocrine carcinoma, is the best-characterized gynecologic neuroendocrine malignancy and is frequently associated with high-risk human papillomavirus infection. In contrast, neuroendocrine neoplasms of the endometrium, ovary, vagina and vulva are much rarer, and available evidence is largely based on small retrospective series and case reports. This rarity has contributed to inconsistent terminology, limited molecular characterization and the absence of site-specific therapeutic standards [3,5].

A major diagnostic challenge is that neuroendocrine marker expression does not necessarily indicate a true neuroendocrine neoplasm. Synaptophysin, chromogranin A, CD56 and INSM1 are useful markers, but focal or aberrant expression may be encountered in conventional gynecologic carcinomas [1,2,6]. Overinterpretation of limited marker positivity may lead to overdiagnosis of neuroendocrine carcinoma and inappropriate treatment intensification, whereas failure to recognize a true neuroendocrine carcinoma or a clinically significant neuroendocrine component may result in underestimation of tumor aggressiveness [1,7].

The biological meaning of neuroendocrine differentiation is also heterogeneous. In some tumors, it reflects a distinct lineage or phenotype, as in well-differentiated ovarian neuroendocrine tumors or HPV-associated cervical neuroendocrine carcinoma. In others, it may represent divergent differentiation within a conventional carcinoma, a component of a mixed neoplasm, or a manifestation of tumor plasticity and dedifferentiation. Emerging molecular data suggest that neuroendocrine differentiation should be interpreted in relation to site-specific oncogenic pathways, including HPV-associated carcinogenesis, TP53-abnormal high-grade tumors, mismatch repair deficiency, *POLE*-mutated endometrial carcinoma, and SWI/SNF-deficient mimics [3,8,9,10,11].

From a clinical perspective, distinguishing incidental neuroendocrine immunophenotype from clinically meaningful neuroendocrine differentiation is essential. Poorly differentiated neuroendocrine carcinomas may require aggressive multimodal treatment and systemic regimens extrapolated from small-cell lung carcinoma or extrapulmonary neuroendocrine carcinoma. By contrast, conventional gynecologic carcinomas with only focal neuroendocrine marker expression are generally managed according to their primary histotype, unless additional morphologic, immunohistochemical or molecular evidence supports a true neuroendocrine phenotype [4,6,10,12].

This narrative review examines neuroendocrine differentiation in tumors of the female genital tract from a clinicopathologic and oncologic perspective. Rather than providing a catalog of rare entities, it aims to clarify the spectrum of neuroendocrine differentiation, define practical diagnostic boundaries, summarize site-specific patterns, and discuss molecular, prognostic, and therapeutic implications.

## 2. Search Strategy and Scope of the Review

This narrative review was designed to provide a clinicopathologic and oncologic overview of neuroendocrine differentiation in tumors of the female genital tract. Given the rarity of these tumors, the heterogeneity of published data, and the predominance of retrospective studies, small case series, and case reports, the review was not intended as a systematic review or meta-analysis.

A literature search was performed using PubMed/MEDLINE, Scopus, Web of Science, and Google Scholar. Search terms included combinations of: “neuroendocrine differentiation”, “female genital tract”, “gynecologic tumors”, “gynecologic cancer”, “neuroendocrine carcinoma”, “small cell neuroendocrine carcinoma”, “large cell neuroendocrine carcinoma”, “cervical neuroendocrine carcinoma”, “endometrial neuroendocrine carcinoma”, “ovarian neuroendocrine tumor”, “ovarian carcinoid”, “vaginal neuroendocrine carcinoma”, “vulvar neuroendocrine carcinoma”, “mixed neuroendocrine carcinoma”, “INSM1”, “synaptophysin”, “chromogranin A” and “CD56”. Additional targeted searches were performed for relevant differential diagnoses and molecular contexts, including “small cell carcinoma of the ovary hypercalcemic type”, “dedifferentiated endometrial carcinoma”, “SWI/SNF deficiency”, “HPV-associated neuroendocrine carcinoma”, “MMR deficiency”, “*POLE* mutation”, and “neuroendocrine transformation”.

Eligible literature included original studies, retrospective cohorts, case series, selected case reports, review articles, consensus documents, and classification references. Priority was given to publications addressing morphology, immunohistochemistry, molecular alterations, differential diagnosis, prognosis, or treatment. Because neuroendocrine neoplasms of the vagina and vulva are exceptionally rare, selected case reports were included when they provided relevant diagnostic or clinicopathologic information.

The review focused on primary tumors of the cervix, endometrium, ovary, vagina and vulva showing neuroendocrine differentiation or neuroendocrine marker expression. Metastatic neuroendocrine neoplasms involving gynecologic organs were discussed only when relevant to differential diagnosis. Studies that focused exclusively on neuroendocrine neoplasms of non-gynecologic sites were excluded, except when they provided a conceptual, molecular, or therapeutic context applicable to gynecologic neuroendocrine carcinomas.

Data were synthesized qualitatively and organized around classification, diagnostic interpretation, site-specific patterns, molecular features, prognostic significance and therapeutic implications. Particular emphasis was placed on distinguishing incidental neuroendocrine marker expression from clinically meaningful neuroendocrine differentiation.

## 3. Defining Neuroendocrine Differentiation: A Diagnostic and Biological Spectrum

The central difficulty raised by neuroendocrine differentiation in female genital tract tumors is that the same immunophenotypic signal may correspond to different biological realities. In one tumor, focal synaptophysin or CD56 expression may represent an incidental finding without major clinical significance; in another, a similar marker profile may support the diagnosis of an aggressive, poorly differentiated neuroendocrine carcinoma. For this reason, neuroendocrine differentiation is best understood as a spectrum rather than as a single diagnostic category [6,10].

At one end of this spectrum is incidental neuroendocrine marker expression, defined by focal, weak or scattered positivity for neuroendocrine markers in a tumor that otherwise shows conventional gynecologic morphology. Such cases may generate diagnostic uncertainty but do not necessarily imply neuroendocrine lineage or neuroendocrine carcinoma-like behavior. Isolated CD56 expression, limited synaptophysin positivity or scattered chromogranin-positive cells should therefore be interpreted cautiously, particularly when architectural and cytologic features of neuroendocrine differentiation are absent [6,10,13].

An intermediate category includes carcinomas with neuroendocrine differentiation, in which neuroendocrine morphology and immunophenotype are both present but may be incomplete, focal or heterogeneously distributed. These tumors occupy a gray zone between conventional carcinoma with marker expression and overt neuroendocrine carcinoma. In such cases, the diagnostic challenge is not simply whether a neuroendocrine marker is positive but whether the overall pattern supports a biologically meaningful neuroendocrine phenotype [10,13].

A further category is represented by mixed neuroendocrine and non-neuroendocrine tumors, in which morphologically distinct components coexist within the same neoplasm. These tumors are clinically relevant because the neuroendocrine component may influence prognosis and treatment, especially when it is poorly differentiated. Their identification requires more than immunohistochemical heterogeneity; it requires recognition of separate morphologic components, ideally supported by component-specific immunophenotypic findings [9,13,14].

At the most definitive end of the spectrum are bona fide neuroendocrine neoplasms, including well-differentiated neuroendocrine tumors and poorly differentiated neuroendocrine carcinomas. Well-differentiated neuroendocrine tumors are most relevant in the ovary, where carcinoid tumors may occur as primary neoplasms, often in association with teratomatous or mucinous elements. Poorly differentiated neuroendocrine carcinomas, by contrast, may arise in several gynecologic sites and include small-cell and large-cell neuroendocrine carcinomas. These tumors are usually high-grade malignancies with necrosis, high mitotic activity and aggressive clinical behavior [4,11,13,15].

Across this spectrum, morphology remains the diagnostic anchor. Neuroendocrine architecture may include organoid nesting, trabecular growth, rosette-like structures and peripheral palisading. Small-cell neuroendocrine carcinoma is characterized by scant cytoplasm, nuclear molding, finely granular chromatin, crush artifact, necrosis and brisk mitotic activity. Large-cell neuroendocrine carcinoma shows larger cells with more abundant cytoplasm, prominent nucleoli, organoid or trabecular growth, necrosis and high proliferative activity. These morphologic features determine whether neuroendocrine marker expression should be regarded as diagnostically meaningful or merely incidental [4,6,10,13].

**Figure 1:**
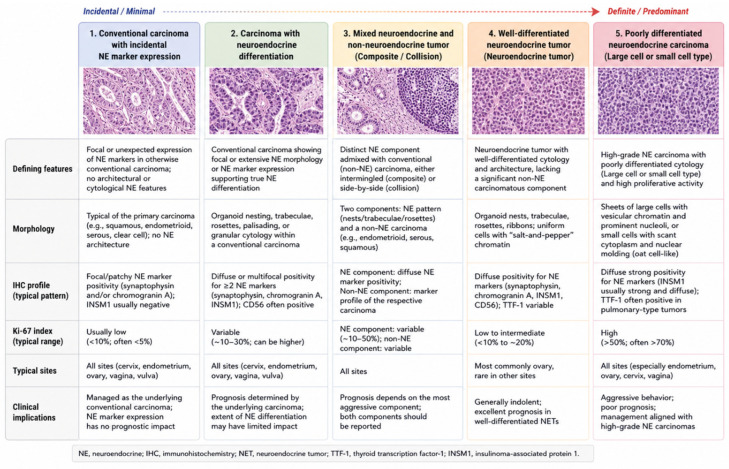
Spectrum of neuroendocrine differentiation in tumors of the female genital tract. The spectrum ranges from incidental neuroendocrine marker expression in conventional carcinomas, through carcinomas with neuroendocrine differentiation and mixed neuroendocrine/non-neuroendocrine tumors, to well-differentiated neuroendocrine tumors and poorly differentiated neuroendocrine carcinomas. The figure emphasizes that morphology remains the diagnostic anchor and that neuroendocrine marker positivity alone is insufficient to define neuroendocrine neoplasia.

This spectrum-based approach has practical consequences. It helps avoid the overdiagnosis of neuroendocrine carcinoma in tumors with limited marker expression, while also reducing the risk of missing a clinically significant neuroendocrine component. The clinicopathologic spectrum of neuroendocrine differentiation in tumors of the female genital tract is summarized in Figure 1, highlighting the transition from incidental neuroendocrine marker expression to bona fide neuroendocrine neoplasia of increasing diagnostic, biologic and therapeutic significance.

## 4. Classification and Diagnostic Terminology

Having defined neuroendocrine differentiation as a diagnostic and biological spectrum, classification requires a clear distinction between immunophenotypic findings, lineage differentiation, and established tumor categories.

### 4.1. From Marker Expression to Diagnostic Category

The most basic distinction is between neuroendocrine marker expression and neuroendocrine neoplasia. Neuroendocrine marker expression refers to immunoreactivity for markers such as synaptophysin, chromogranin A, CD56 or INSM1, without necessarily implying that the tumor belongs to a neuroendocrine lineage. This situation is especially relevant when staining is focal, weak, limited to scattered cells or restricted to a single marker. In such cases, the finding should be interpreted as an immunophenotypic observation rather than as a diagnostic category [6,7,10].

By contrast, neuroendocrine neoplasia implies concordance between morphology and immunophenotype. A tumor should be classified as a neuroendocrine neoplasm only when neuroendocrine architecture or cytology is supported by appropriate marker expression. Thus, terms such as neuroendocrine tumor and neuroendocrine carcinoma should be reserved for defined entities, whereas carcinoma with focal neuroendocrine marker expression or carcinoma with neuroendocrine differentiation may be more appropriate for borderline or incomplete patterns [4,10,13].

The term neuroendocrine carcinoma implies a high-grade malignancy with potential prognostic and therapeutic consequences. It should therefore not be applied to conventional gynecologic carcinomas showing only limited or nonspecific marker expression [6,13].

### 4.2. Well-Differentiated Neuroendocrine Tumors and Poorly Differentiated Neuroendocrine Carcinomas

The principal recognized categories of neuroendocrine neoplasia are well-differentiated neuroendocrine tumors and poorly differentiated neuroendocrine carcinomas. In the female genital tract, well-differentiated neuroendocrine tumors are most relevant in the ovary, where they have traditionally been referred to as ovarian carcinoid tumors. They may occur as pure tumors or in association with mature cystic teratomas or mucinous tumors, and their architecture may include insular, trabecular, mucinous or strumal patterns. When confined to the ovary, these tumors are generally more indolent than poorly differentiated neuroendocrine carcinomas [11,13,16,17].

Poorly differentiated neuroendocrine carcinomas are high-grade epithelial malignancies characterized by neuroendocrine morphology and supportive marker expression. They are usually classified as small-cell neuroendocrine carcinoma or large-cell neuroendocrine carcinoma, based on cytologic and architectural features [4,13,18]. Small-cell neuroendocrine carcinoma is composed of small to intermediate-sized cells with scant cytoplasm, nuclear molding, finely granular chromatin, frequent mitoses and necrosis. Large-cell neuroendocrine carcinoma shows larger cells with more abundant cytoplasm, prominent nucleoli, organoid or trabecular architecture, high mitotic activity and necrosis. Within the female genital tract, poorly differentiated neuroendocrine carcinoma is best established in the cervix [4,5]. In the endometrium, ovary, vagina and vulva, these tumors are much rarer and often pose greater diagnostic difficulty because of overlap with high-grade non-neuroendocrine malignancies and metastatic neuroendocrine carcinomas. A diagnosis of primary gynecologic poorly differentiated neuroendocrine carcinoma should therefore be supported by compatible morphology, appropriate immunophenotype and clinicopathologic exclusion of a more likely extragenital primary [5,13].

### 4.3. Mixed Neuroendocrine and Non-Neuroendocrine Tumors

Mixed neuroendocrine and non-neuroendocrine tumors contain morphologically distinct neuroendocrine and conventional non-neuroendocrine components within the same neoplasm. In the female genital tract, the non-neuroendocrine component may include squamous cell carcinoma, adenocarcinoma, endometrioid carcinoma, serous carcinoma, mucinous carcinoma or other site-specific histotypes.

The diagnosis of a mixed tumor should not be based solely on heterogeneous immunohistochemical staining. Rather, it requires the recognition of separate morphologic components, with immunohistochemistry used to support the identity of the neuroendocrine component. Patchy synaptophysin or CD56 expression within an otherwise uniform conventional carcinoma does not necessarily indicate a mixed neoplasm [9,10,11,14].

When a mixed tumor is identified, both components should be included in the diagnostic wording whenever possible, and the approximate proportion of each should be reported. This is particularly important when the neuroendocrine component is poorly differentiated, as it may influence prognosis and treatment. Mixed tumors are especially relevant in the cervix, where neuroendocrine carcinoma may coexist with HPV-associated squamous cell carcinoma or adenocarcinoma, but they may also occur in the endometrium and ovary [5,9,13,14].

### 4.4. Practical Terminology Across Gynecologic Sites

Although the same broad categories apply across the female genital tract, their practical use differs by site. In the cervix, small-cell and large-cell neuroendocrine carcinomas are the most well-established entities, and any associated squamous or glandular components should be documented. In the endometrium, terminology requires particular caution because neuroendocrine carcinoma may overlap with undifferentiated/dedifferentiated carcinoma, serous carcinoma, carcinosarcoma, and other high-grade tumors. In the ovary, classification must distinguish primary well-differentiated neuroendocrine tumors, poorly differentiated neuroendocrine carcinoma, and metastatic neuroendocrine neoplasms. In the vagina and vulva, primary neuroendocrine carcinomas are exceptionally rare, and diagnostic wording should reflect the need to exclude cervical extension, cutaneous Merkel cell carcinoma-like tumors, or metastatic disease [4,9,10,11,13,19].

For consistency, this review uses the following terminology: neuroendocrine marker expression for focal or isolated marker positivity without convincing morphology; neuroendocrine differentiation for tumors in which marker expression is supported by at least partial neuroendocrine architecture or cytology; well-differentiated neuroendocrine tumor for tumors with well-differentiated neuroendocrine morphology, most often relevant in the ovary; poorly differentiated neuroendocrine carcinoma for high-grade small-cell or large-cell neuroendocrine carcinoma; and mixed neuroendocrine and non-neuroendocrine tumor for neoplasms containing morphologically distinct components.

These definitions provide the terminology required for the next stage of evaluation: the practical recognition of neuroendocrine differentiation in routine diagnostic work.

## 5. Immunohistochemical Assessment: Markers, Interpretation and Diagnostic Pitfalls

The terminology outlined above depends in practice on the careful interpretation of immunohistochemistry. In tumors of the female genital tract, neuroendocrine markers are indispensable for confirming suspected neuroendocrine differentiation, but they can also generate diagnostic error when interpreted without adequate morphologic and clinical correlation.

### 5.1. Core Neuroendocrine Markers

The most widely used markers of neuroendocrine differentiation include synaptophysin, chromogranin A, CD56 and INSM1. These markers differ in sensitivity, specificity and staining pattern, and none should be interpreted in isolation [13].

Synaptophysin is one of the most sensitive neuroendocrine markers and is frequently used as part of the initial diagnostic panel. It typically shows cytoplasmic staining and is often positive in both well-differentiated neuroendocrine tumors and poorly differentiated neuroendocrine carcinomas. However, synaptophysin is not entirely specific, and focal or patchy expression may be encountered in non-neuroendocrine carcinomas. Its diagnostic weight is greatest when staining is diffuse, strong and concordant with neuroendocrine morphology [4,6,20,21,22,23].

Chromogranin A is generally more specific but less sensitive than synaptophysin. It is particularly useful in well-differentiated neuroendocrine tumors, where secretory granules are more abundant. In poorly differentiated neuroendocrine carcinomas, chromogranin A expression may be focal, weak or absent. Therefore, a negative chromogranin A result does not exclude neuroendocrine carcinoma when morphology and other markers are supportive [7,21,22,24].

CD56 is sensitive but relatively nonspecific. Its broad expression pattern limits its diagnostic value, particularly when it is the only positive neuroendocrine marker. Isolated CD56 positivity should not establish a diagnosis of neuroendocrine differentiation or neuroendocrine carcinoma. Its role is best regarded as supportive within a broader panel.

INSM1 has emerged as a useful nuclear marker of neuroendocrine differentiation, particularly in poorly differentiated neuroendocrine carcinomas. Its nuclear staining pattern may be easier to interpret than cytoplasmic markers, especially in small biopsies or crushed specimens. Nevertheless, INSM1 should also be interpreted in relation to morphology and the overall immunohistochemical profile [18,22,25].

In routine practice, convincing neuroendocrine differentiation is best supported by concordant morphology and expression of more than one neuroendocrine marker, particularly when staining is diffuse and strong. Conversely, focal positivity for a single marker in a tumor with conventional morphology should be reported cautiously and should not drive diagnostic reclassification [7,10]. The practical utility and major interpretive limitations of the core neuroendocrine and ancillary non-neuroendocrine markers are summarized in Table 1.

### 5.2. Proliferation, Epithelial Lineage and Site-Directed Markers

Neuroendocrine markers should be interpreted together with markers that establish tumor lineage, proliferation and site of origin. Ki-67 is useful for assessing proliferative activity, especially in distinguishing well-differentiated neuroendocrine tumors from poorly differentiated neuroendocrine carcinomas in the appropriate context. However, Ki-67 does not establish neuroendocrine lineage; it reflects proliferation, not differentiation. Epithelial markers, including broad-spectrum cytokeratins and epithelial membrane antigen, are important for confirming carcinoma, particularly in small biopsies or poorly differentiated tumors. This is essential before classifying a lesion as poorly differentiated neuroendocrine carcinoma, because lymphoma, melanoma, sarcoma and other small round cell tumors may enter the differential diagnosis [2,13,26]. Site-directed markers should be selected according to anatomic location and differential diagnosis. PAX8, ER, PR and WT1 may help support Müllerian or ovarian/endometrial origin in appropriate settings, although none is definitive in isolation. In cervical tumors, p16 and HPV testing are particularly important; diffuse block-type p16 expression and high-risk HPV detection may support primary HPV-associated cervical neuroendocrine carcinoma when morphology and clinical findings are concordant. In vulvar lesions, CK20, neurofilament and Merkel cell polyomavirus may help evaluate Merkel cell carcinoma-like tumors [5,26,27,28]. Markers used to investigate metastatic origin include TTF-1, CDX2, SATB2 and CK20, among others. TTF-1 may support pulmonary origin, while CDX2 and SATB2 may suggest intestinal differentiation, particularly in ovarian tumors where metastatic gastrointestinal neuroendocrine neoplasm is a major consideration. However, these markers should not be interpreted in isolation, because expression patterns may overlap and site assignment requires clinicoradiologic correlation [11,29,30] (Table 1).

### 5.3. Molecularly Relevant Immunohistochemical Markers

Some immunohistochemical markers provide molecular context and may help separate true neuroendocrine carcinoma from important mimics. In endometrial tumors, p53 and mismatch repair proteins are particularly relevant because molecular classification is central to current endometrial carcinoma interpretation. Abnormal p53 expression may support a copy-number-high or serous-like pathway, whereas loss of mismatch repair proteins indicates MMR deficiency and may have therapeutic implications [10,31,32,33]. SWI/SNF-related markers may be useful when undifferentiated or dedifferentiated endometrial carcinoma or small-cell carcinoma of the ovary, hypercalcemic type, is considered in the differential diagnosis. Loss of SMARCA4, SMARCB1 or other SWI/SNF complex proteins may redirect the diagnosis away from neuroendocrine carcinoma and toward a specific non-neuroendocrine mimic. These markers should be used selectively, guided by morphology and clinical context, rather than applied indiscriminately [10,20,34,35] (Table 1).

### 5.4. Practical Interpretation of Staining Patterns

The diagnostic significance of immunohistochemistry depends not only on which markers are positive, but also on how they are expressed. Reports should, when relevant, describe whether staining is focal, patchy, or diffuse, weak or strong, limited to scattered cells or present in a morphologically distinct component, and whether one or multiple markers are positive [29].

Diffuse staining for multiple neuroendocrine markers in a tumor with compatible morphology strongly supports neuroendocrine differentiation. In contrast, weak or focal staining, isolated CD56 positivity or scattered positive cells in a tumor with conventional morphology should be interpreted cautiously. Even multiple-marker positivity should not override morphology if the architecture and cytology are entirely non-neuroendocrine. There is no universally applicable percentage threshold that reliably separates incidental neuroendocrine marker expression from clinically meaningful neuroendocrine differentiation across all gynecologic sites. Therefore, rigid cut-offs should be avoided unless required by a specific classification context. A more practical approach is to assess whether staining is concordant with morphology, whether it highlights a distinct component, and whether the immunophenotype is consistent across the tumor [13,21,22]. A pragmatic diagnostic workflow begins with morphology. If neuroendocrine differentiation is suspected, a core panel may include synaptophysin, chromogranin A and INSM1, with CD56 used only as a supportive marker. Epithelial markers and site-directed markers should then be added according to the differential diagnosis. The final interpretation should distinguish between incidental marker expression, carcinoma with neuroendocrine differentiation, mixed neuroendocrine/non-neuroendocrine tumor, well-differentiated neuroendocrine tumor, and poorly differentiated neuroendocrine carcinoma [36].

## 6. Site-Specific Patterns of Neuroendocrine Differentiation in the Female Genital Tract

**Figure 2:**
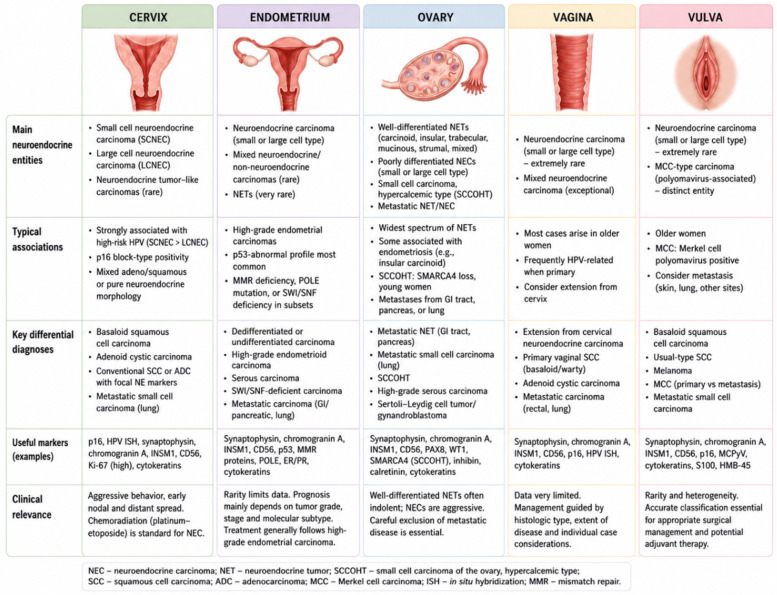
Site-specific landscape of neuroendocrine differentiation in the female genital tract. Overview of the main neuroendocrine entities, typical clinicopathologic associations, key differential diagnoses, useful immunohistochemical markers and clinical implications across the cervix, endometrium, ovary, vagina and vulva. The figure emphasizes that neuroendocrine differentiation has different diagnostic and clinical significance depending on anatomic site and should always be interpreted in a morphologic and clinicopathologic context.

The principles outlined above apply across the female genital tract, but their practical interpretation varies substantially by anatomic site. Neuroendocrine differentiation in the cervix, endometrium, ovary, vagina and vulva does not represent a single biological entity. Each site has distinct tumor types, molecular associations, diagnostic mimics and clinical implications (Figure 2) [13,36].

### 6.1. Cervix

The cervix is the best-established site for primary gynecologic neuroendocrine carcinoma. The main entities are small-cell neuroendocrine carcinoma and large-cell neuroendocrine carcinoma, although mixed tumors with squamous or glandular components are also encountered. Cervical neuroendocrine carcinomas are uncommon but clinically important because they are typically more aggressive than conventional cervical squamous cell carcinoma or adenocarcinoma [5,13,24,37].

Small-cell neuroendocrine carcinoma is the prototypical cervical neuroendocrine malignancy. It usually shows scant cytoplasm, nuclear molding, finely granular chromatin, necrosis, brisk mitotic activity, and frequent crush artifacts. Large-cell neuroendocrine carcinoma is less common and shows larger cells with more abundant cytoplasm, prominent nucleoli, and neuroendocrine architecture such as nesting, trabecular growth, or rosette-like structures [5,9].

A defining feature of cervical neuroendocrine carcinoma is its frequent association with high-risk human papillomavirus. Diffuse block-type p16 expression and HPV detection support a primary cervical origin when morphology and clinical findings are concordant. The presence of an associated HPV-related squamous cell carcinoma or adenocarcinoma may further support primary cervical origin and suggests divergent differentiation within an HPV-driven neoplasm [5,24,38,39]. However, p16 expression alone is insufficient to prove origin, and metastatic small-cell carcinoma, particularly from the lung, should be considered when clinical or morphologic features are atypical. Limited synaptophysin or CD56 positivity in an otherwise typical squamous cell carcinoma or adenocarcinoma should not automatically lead to reclassification. Conversely, a high-grade tumor with small-cell or large-cell morphology should prompt a neuroendocrine panel even when a conventional component is also present [38,39].

From an oncologic perspective, cervical neuroendocrine carcinoma is associated with early lymphovascular invasion, nodal involvement and distant metastasis. Even apparently localized tumors may relapse systemically, which explains why recognition of this phenotype has direct implications for staging, imaging and treatment planning [4,40,41].

### 6.2. Endometrium

In the endometrium, neuroendocrine differentiation is less well defined and usually arises in the setting of high-grade carcinoma, mixed histology or undifferentiated morphology. True primary endometrial neuroendocrine carcinoma is rare, and reported tumors often contain additional components such as endometrioid carcinoma, serous carcinoma, undifferentiated carcinoma or carcinosarcoma [36,42].

The main diagnostic issue is distinguishing clinically meaningful neuroendocrine differentiation from focal marker expression in high-grade endometrial carcinoma. Endometrioid and serous carcinomas may show limited neuroendocrine marker positivity without fulfilling criteria for neuroendocrine carcinoma. Conversely, a poorly differentiated tumor with organoid architecture, necrosis, high mitotic activity and diffuse neuroendocrine marker expression may represent true neuroendocrine carcinoma or a mixed carcinoma with a neuroendocrine component [10,36,42].

Endometrial tumors also require integration with molecular classification. p53-abnormal high-grade tumors, mismatch repair-deficient carcinomas, *POLE*-mutated tumors and SWI/SNF-deficient undifferentiated/dedifferentiated carcinomas may all enter the diagnostic discussion. In particular, undifferentiated and dedifferentiated carcinoma may mimic neuroendocrine carcinoma morphologically, while SWI/SNF loss may redirect the diagnosis toward a non-neuroendocrine mimic [10,43,44].

Limited biopsy material is a frequent problem. A biopsy may sample only the neuroendocrine-like component or only the conventional component of a heterogeneous tumor. Therefore, reports should specify whether neuroendocrine features are focal or diffuse, whether a distinct component is present, and how the findings relate to the underlying endometrial carcinoma type and molecular context [42,43].

Clinically, true endometrial poorly differentiated neuroendocrine carcinoma appears aggressive, although available data remain limited. By contrast, the prognostic significance of focal neuroendocrine marker expression in otherwise conventional endometrial carcinoma is uncertain and should not be overstated [10,42].

### 6.3. Ovary

The ovary shows the broadest neuroendocrine spectrum among female genital tract sites. Ovarian lesions may include well-differentiated primary neuroendocrine tumors, poorly differentiated neuroendocrine carcinomas, mixed tumors and metastatic neuroendocrine neoplasms from extragenital sites. Distinguishing these categories is essential because they differ substantially in biology and management.

Well-differentiated ovarian neuroendocrine tumors have traditionally been referred to as ovarian carcinoid tumors [36,45]. They may show insular, trabecular, mucinous or strumal patterns and often occur in association with mature cystic teratomas or mucinous tumors. When confined to the ovary, they are generally more indolent than poorly differentiated neuroendocrine carcinoma. However, accurate classification requires the exclusion of metastatic neuroendocrine tumors, particularly from gastrointestinal or pancreatic sites [45,46,47].

Features supporting primary ovarian origin include unilateral disease, association with teratomatous or mucinous elements and the absence of an extragenital primary. Bilaterality, ovarian surface involvement, multinodular growth, extraovarian disease or a known history of gastrointestinal or pancreatic neuroendocrine tumor should raise concern for metastasis. Immunohistochemistry may help, but no single marker is definitive; interpretation requires clinical and radiologic correlation [11,36,47].

Poorly differentiated ovarian neuroendocrine carcinoma is rare and aggressive. It may show small-cell or large-cell morphology and can overlap with other high-grade ovarian malignancies. A particularly important differential diagnosis is small-cell carcinoma of the ovary, hypercalcemic type, which is not a conventional neuroendocrine carcinoma despite its name and small-cell morphology. Recognition of this entity is critical because it is associated with SWI/SNF alterations, especially SMARCA4 loss, and requires different diagnostic framing [35,48,49].

Ovarian mixed tumors may contain well-differentiated neuroendocrine elements associated with teratomatous or mucinous tumors, or poorly differentiated neuroendocrine carcinoma associated with epithelial malignancy. The clinical meaning of the neuroendocrine component depends strongly on whether it is well differentiated or poorly differentiated, as well as on stage and metastatic pattern [11,15,50].

### 6.4. Vagina and Vulva

Vaginal and vulvar neuroendocrine carcinomas are exceptionally rare, and most available evidence comes from case reports or small series. Because of this rarity, diagnosis requires careful exclusion of extension from adjacent organs and metastatic disease. In the vagina, a neuroendocrine carcinoma may represent a true primary tumor, direct cervical extension, recurrence of cervical carcinoma or metastasis from an extragenital site. Clinical examination, imaging and assessment of the cervix are therefore essential [13,19,51,52].

When a primary vaginal neuroendocrine carcinoma is considered, the diagnostic approach should follow the same integrated principles used elsewhere: compatible morphology, expression of neuroendocrine markers, and exclusion of mimics. p16 and HPV testing may support an HPV-associated mucosal origin in selected cases, but HPV-related markers must be interpreted in relation to anatomic findings because cervical primaries may show similar results [52,53].

The vulva poses additional complexity because it lies at the interface of mucosal gynecologic and cutaneous pathology. Primary vulvar neuroendocrine carcinoma is rare, and the differential diagnosis includes Merkel cell carcinoma-like tumors, basaloid squamous cell carcinoma, melanoma, adnexal tumors, and metastatic neuroendocrine carcinoma. CK20, neurofilament, Merkel cell polyomavirus, p16/HPV testing, squamous markers, and melanocytic markers may be required depending on morphology [19,51,54].

As in other sites, neuroendocrine marker expression alone is insufficient for diagnostic reclassification. This is particularly relevant in poorly differentiated vulvar squamous lesions with basaloid morphology and limited marker expression. Because clinical data are sparse and treatment is not standardized, precise classification and multidisciplinary correlation are especially important for vaginal and vulvar tumors [52,54].

## 7. The Gray Zone: Incidental Immunophenotype Versus Clinically Meaningful Neuroendocrine Differentiation

The more difficult cases are those showing limited, partial, or discordant neuroendocrine features. These tumors occupy a gray zone in which the central question is whether neuroendocrine marker expression represents an incidental immunophenotypic finding or a biologically meaningful component of the neoplasm [6,10,22].

### 7.1. Incidental Marker Expression and Partial Neuroendocrine Differentiation

Incidental neuroendocrine marker expression refers to focal, weak, or scattered positivity for one or more neuroendocrine markers in a tumor that otherwise retains conventional morphology. This may be encountered in endometrioid carcinoma, serous carcinoma, poorly differentiated squamous cell carcinoma, ovarian epithelial carcinoma, and other gynecologic malignancies. In such cases, the finding is best regarded as an immunohistochemical observation rather than evidence of a separate neuroendocrine neoplasm [6,10,55].

Features favoring an incidental interpretation include staining limited to rare cells, weak or patchy staining, positivity for only one marker, isolated CD56 expression, and the absence of neuroendocrine architecture or cytology. These tumors should generally retain their conventional histologic classification. If the finding is mentioned, wording such as “focal neuroendocrine marker expression” is preferable to “neuroendocrine carcinoma” [10,22,55].

A more challenging situation arises when marker expression is accompanied by partial morphologic features suggestive of neuroendocrine differentiation, such as focal organoid nesting, trabecular growth, rosette-like structures, nuclear molding or high-grade cytology. These tumors may represent divergent differentiation within a conventional carcinoma, an under-sampled mixed tumor or a poorly differentiated carcinoma with overlapping features. In such cases, the term “carcinoma with neuroendocrine differentiation” may be appropriate, provided that the report describes the extent of the finding and its limitations [10,13,22].

### 7.2. Criteria Favoring Clinically Meaningful Differentiation

Clinically meaningful neuroendocrine differentiation is more likely when morphology and immunophenotype are concordant. The strongest support comes from a tumor that shows classic small-cell or large-cell neuroendocrine morphology, together with diffuse expression of multiple neuroendocrine markers. INSM1 positivity may strengthen the interpretation when present in the appropriate morphologic context, but it should not override morphology [21].

Several findings support biological significance: a morphologically distinct neuroendocrine component, diffuse multi-marker expression, high mitotic activity and necrosis, and a metastatic pattern compatible with neuroendocrine carcinoma. Site-specific context may also be important, such as HPV-associated cervical origin or an ovarian tumor lacking features of metastatic gastrointestinal or pancreatic neuroendocrine tumor. No universally applicable percentage threshold reliably separates incidental neuroendocrine marker expression from clinically meaningful neuroendocrine differentiation across all gynecologic sites. A small but morphologically distinct poorly differentiated neuroendocrine component may be more relevant than extensive but nonspecific marker expression in a conventional carcinoma. Therefore, the relationship between staining and morphology is more important than a rigid numerical cut-off [46,56,57].

### 7.3. Reporting Gray-Zone Cases

Reporting should communicate both the finding and the degree of diagnostic certainty. In a tumor with conventional morphology and focal marker expression, the final diagnosis should remain the conventional histotype, with a comment such as: “Focal expression of neuroendocrine markers is present; however, the morphologic features do not support a diagnosis of neuroendocrine carcinoma.” In a tumor with partial morphologic and immunophenotypic support, wording such as “carcinoma with neuroendocrine differentiation” may be used, accompanied by a comment specifying whether the features are focal, patchy, biopsy-limited or insufficient for definitive classification as neuroendocrine carcinoma. When a distinct component is present, the tumor should be reported as mixed, with both components identified and the approximate extent of the neuroendocrine component documented when possible [3,36].

This careful wording has clinical consequences. Overdiagnosis may lead to unnecessary treatment intensification and inaccurate prognostic counseling, whereas underrecognition of a true neuroendocrine carcinoma or significant neuroendocrine component may underestimate metastatic risk. Gray-zone cases should therefore be approached as interpretive diagnoses requiring the integration of morphology, immunohistochemistry, site-specific context, and clinical information, rather than as automatic consequences of marker positivity [3].

Among these gray-zone lesions, mixed neuroendocrine and non-neuroendocrine tumors deserve separate discussion because they represent a morphologically identifiable form of tumor heterogeneity rather than merely ambiguous immunoreactivity [36,58]. Practical diagnostic wording for common interpretive scenarios is summarized in Table 2, with emphasis on avoiding both overdiagnosis of neuroendocrine carcinoma and underrecognition of clinically meaningful neuroendocrine differentiation.

## 8. Mixed Neuroendocrine and Non-Neuroendocrine Tumors

Unlike gray-zone cases with limited or ambiguous marker expression, mixed tumors contain a recognizable neuroendocrine component and a conventional non-neuroendocrine component within the same neoplasm. This distinction is important because the neuroendocrine component, particularly when poorly differentiated, may influence prognosis and treatment.

### 8.1. Definition and Patterns Across Gynecologic Sites

The diagnosis of a mixed neuroendocrine and non-neuroendocrine tumor requires morphologic recognition of distinct components. It should not be based solely on patchy expression of synaptophysin, chromogranin A, CD56 or INSM1 within an otherwise uniform carcinoma. Immunohistochemistry is used to support the identity of the neuroendocrine component, but the defining feature is morphologic separateness. In the cervix, mixed tumors are most often composed of poorly differentiated neuroendocrine carcinoma associated with squamous cell carcinoma or adenocarcinoma. The presence of high-risk HPV in both components may support divergent differentiation from a shared HPV-driven precursor, and recognition of the conventional component may help establish primary cervical origin [3,5,59,60].

In the endometrium, mixed tumors are more difficult to classify because high-grade endometrial carcinomas are often heterogeneous. A neuroendocrine component may coexist with endometrioid carcinoma, serous carcinoma, undifferentiated carcinoma or carcinosarcoma-like elements. In such cases, the key issue is whether the neuroendocrine area represents a true distinct component or neuroendocrine marker expression within a broader high-grade carcinoma [3,42].

In the ovary, mixed tumors may contain well-differentiated neuroendocrine elements associated with teratomatous or mucinous tumors or poorly differentiated neuroendocrine carcinoma associated with epithelial malignancy. These scenarios have different biological implications and should not be grouped together without specifying the type and grade of the neuroendocrine component. Mixed tumors of the vagina and vulva are rare, and diagnosis in these sites requires careful exclusion of extension or metastasis [11,50].

### 8.2. Biological and Clinical Significance

Mixed tumors raise important pathogenetic questions. In some cases, neuroendocrine and non-neuroendocrine components may arise from a common precursor and diverge during tumor evolution. This is particularly plausible in HPV-associated cervical tumors. In other settings, mixed morphology may reflect divergent differentiation within a high-grade carcinoma or the emergence of a neuroendocrine subclone during tumor progression [5,41,60,61].

Molecular comparison of components may clarify clonal relationships, especially if shared driver alterations are identified. However, such data remain limited in gynecologic tumors, and most diagnoses still rely on morphology, immunohistochemistry and clinicopathologic correlation. The immediate clinical value of molecular analysis is to refine classification, identify actionable features and determine whether the neuroendocrine component shares the molecular background of the associated carcinoma [41,60].

The clinical significance of a mixed tumor depends on the type, grade and extent of the neuroendocrine component, as well as on stage and metastatic pattern. A poorly differentiated neuroendocrine carcinoma component may drive aggressive behavior and influence systemic therapy, even when it represents only part of the tumor. By contrast, a well-differentiated neuroendocrine component in an ovarian teratoma or mucinous tumor has different implications and should not be equated with high-grade neuroendocrine carcinoma [11,62,63].

## 9. Differential Diagnosis and Metastatic Mimics

**Figure 3:**
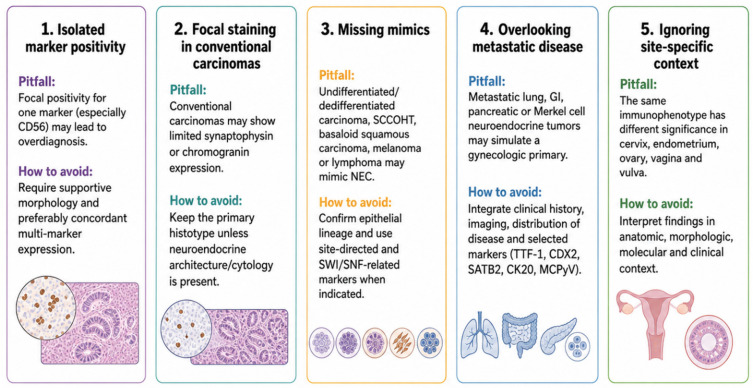
Common diagnostic pitfalls in interpreting neuroendocrine differentiation in tumors of the female genital tract. The figure highlights frequent sources of misinterpretation, including isolated neuroendocrine marker positivity, focal staining in otherwise conventional carcinomas, non-neuroendocrine mimics, metastatic neuroendocrine tumors and failure to account for site-specific context. Accurate diagnosis requires correlation of morphology, immunohistochemical staining pattern, anatomic site, molecular findings, clinical history and imaging, rather than reliance on neuroendocrine marker positivity alone.

Because primary neuroendocrine neoplasms of the female genital tract are rare, mimics and metastases must be considered carefully before assigning a definitive diagnosis. This is particularly important in the ovary, vagina, and vulva, where metastatic disease or extension from adjacent anatomic sites may closely simulate a primary neuroendocrine tumor. Differential diagnosis should integrate morphology, immunohistochemistry, molecular findings, clinical history, and imaging, rather than relying on a single marker or isolated histologic feature (Figure 3) [3,11,47,52].

### 9.1. Conventional Gynecologic Carcinomas and High-Grade Epithelial Mimics

One of the most frequent diagnostic pitfalls is a conventional gynecologic carcinoma with focal neuroendocrine marker expression. Endometrioid carcinoma, serous carcinoma, poorly differentiated squamous cell carcinoma, HPV-associated adenocarcinoma, and ovarian epithelial carcinomas may occasionally express synaptophysin, chromogranin A, CD56, or INSM1. If the morphology remains conventional, such tumors should not be reclassified as neuroendocrine carcinoma solely on the basis of marker positivity [10,22,25].

High-grade epithelial tumors may further complicate the differential diagnosis because solid growth, necrosis, high mitotic activity and marked cytologic atypia can resemble poorly differentiated neuroendocrine carcinoma. Basaloid squamous cell carcinoma, particularly in the cervix and vulva, may show small, crowded cells, a high nuclear-to-cytoplasmic ratio, and necrosis. p40 or p63 expression, squamous differentiation, and an associated intraepithelial squamous lesion may support squamous lineage, although p16 can be positive in both HPV-associated squamous carcinoma and cervical neuroendocrine carcinoma [36,38,64,65].

Other epithelial mimics include poorly differentiated adenocarcinoma, serous carcinoma, and carcinosarcoma. These tumors may show high-grade morphology and occasional neuroendocrine marker expression, but their classification should be based on the dominant morphologic pattern, lineage-specific markers, and associated precursor or conventional components. A broad panel is often required in small biopsies, especially when morphology is limited or a crush artifact is present [3,6,10,29].

### 9.2. Undifferentiated Tumors and Small Round Cell Mimics

Undifferentiated and dedifferentiated endometrial carcinomas are among the most important mimics of endometrial neuroendocrine carcinoma. They may show diffuse solid growth, high-grade cytology, necrosis, and loss of glandular differentiation. In limited samples, they can resemble poorly differentiated neuroendocrine carcinoma, particularly when the tumor is monotonous or poorly cohesive. SWI/SNF abnormalities, including loss of SMARCA4, SMARCB1, ARID1A, or ARID1B, depending on context, may support undifferentiated/dedifferentiated carcinoma rather than true neuroendocrine carcinoma [10,36].

Small-cell carcinoma of the ovary, hypercalcemic type, is a critical ovarian mimic. It should be considered especially in young patients with ovarian tumors showing small-cell or undifferentiated morphology, particularly if hypercalcemia is present. Demonstration of SMARCA4/BRG1 loss can redirect the diagnosis and prevent inappropriate classification as ovarian neuroendocrine carcinoma [48,49].

Other small round cell mimics include lymphoma, melanoma, sarcoma-like tumors, germ cell tumors and sex cord–stromal tumors. These are particularly relevant in small biopsies from the cervix, vagina or vulva. Leukocyte markers can exclude lymphoma; melanocytic markers such as SOX10, S100, HMB45 and Melan-A can identify melanoma; and appropriate germ cell or sex cord–stromal markers should be selected according to morphology and patient context. Confirming epithelial lineage with cytokeratins and epithelial membrane antigen is an essential step before diagnosing poorly differentiated neuroendocrine carcinoma [3,29,36].

### 9.3. Metastatic Neuroendocrine Neoplasms

Metastatic neuroendocrine neoplasms are a major consideration in gynecologic organs, especially the ovary, vagina, and vulva. Potential primary sites include the lung, gastrointestinal tract, pancreas, appendix, and skin/Merkel cell carcinoma. In some cases, a gynecologic lesion may be the first manifestation of an occult extragenital primary. The ovary is a particularly important site for metastatic well-differentiated neuroendocrine tumors, especially from gastrointestinal or pancreatic primaries. Bilateral ovarian involvement, multinodular growth, surface implants, extraovarian disease, and the absence of teratomatous or mucinous ovarian components should raise suspicion for metastasis. Conversely, unilateral disease associated with a mature teratoma or a mucinous neoplasm supports primary ovarian origin, although this is not definitive [3,11,47,66].

Metastatic pulmonary small-cell carcinoma may involve the cervix, vagina, ovary or vulva and closely resemble primary gynecologic small-cell neuroendocrine carcinoma. Clinical history and imaging are essential [42,67]. TTF-1 may support pulmonary origin, but it is not entirely specific and should not be interpreted alone. Similarly, CDX2 and SATB2 may support intestinal origin, particularly in ovarian tumors, but must be interpreted with morphology and disease distribution [29,42,68,69].

Merkel cell carcinoma is especially relevant in vulvar lesions. Primary cutaneous Merkel cell carcinoma, metastatic Merkel cell carcinoma and Merkel cell carcinoma-like vulvar tumors may all show neuroendocrine morphology and marker expression. CK20, neurofilament, Merkel cell polyomavirus and p16/HPV testing may be useful, but final classification depends on the anatomic distribution of the lesion and the distinction between mucosal, cutaneous and metastatic disease [27,54].

## 10. Molecular Features and Biological Insights

Molecular data provide an important biological context for interpreting neuroendocrine differentiation in female genital tract tumors. They may help clarify tumor origin, identify mimics, support the assessment of clonal relationships in mixed tumors, and reveal potentially actionable alterations. However, the molecular landscape of gynecologic tumors with neuroendocrine differentiation remains incompletely defined, largely because most available studies include small cohorts, use heterogeneous terminology, and have limited site-specific sequencing data [13,57,61,70].

### 10.1. Site-Specific Molecular Contexts

The best-established molecular association is the relationship between cervical neuroendocrine carcinoma and high-risk human papillomavirus. Cervical small-cell and large-cell neuroendocrine carcinomas are frequently HPV-associated, and diffuse p16 expression is often used as a surrogate marker, although HPV testing provides more direct evidence. The coexistence of neuroendocrine carcinoma with squamous cell carcinoma or adenocarcinoma in the cervix supports the concept of divergent differentiation within a shared HPV-driven neoplastic process [5,36,64].

In endometrial tumors, neuroendocrine differentiation should be interpreted within the molecular framework of endometrial carcinoma. p53-abnormal high-grade tumors, mismatch repair-deficient carcinomas and *POLE*-mutated tumors may show high-grade or heterogeneous morphology, and neuroendocrine features may coexist with these molecular contexts. MMR deficiency may have therapeutic relevance, particularly in advanced or recurrent disease, while *POLE* mutation may modify prognostic interpretation. p53-abnormal status may support a high-grade serous-like or copy-number-high pathway, but it does not by itself define neuroendocrine differentiation [1,13,71,72,73].

In the ovary, molecular interpretation depends strongly on tumor category. Well-differentiated ovarian neuroendocrine tumors, particularly those associated with teratomatous elements, differ biologically from poorly differentiated ovarian neuroendocrine carcinomas and from metastatic neuroendocrine tumors. The distinction from small-cell carcinoma of the ovary, hypercalcemic type, is especially important because the latter is strongly associated with *SMARCA4* inactivation and should not be classified as a conventional neuroendocrine carcinoma [13,46,62].

### 10.2. SWI/SNF-Deficient Mimics and Diagnostic Refinement

SWI/SNF complex alterations are particularly relevant because several SWI/SNF-deficient tumors may mimic neuroendocrine carcinoma. Undifferentiated and dedifferentiated endometrial carcinomas may show solid growth, high-grade cytology and reduced epithelial differentiation, creating overlap with poorly differentiated neuroendocrine carcinoma. Similarly, small-cell carcinoma of the ovary, hypercalcemic type, may resemble ovarian neuroendocrine carcinoma morphologically but is genetically and biologically distinct [35,43].

Loss of SMARCA4, SMARCB1 or other SWI/SNF-related proteins can redirect the diagnosis toward a specific non-neuroendocrine entity. This is particularly useful when neuroendocrine marker expression is limited, discordant or not supported by classic morphology. In such cases, molecularly informed immunohistochemistry does not replace morphology, but it may prevent misclassification and inappropriate grouping with true neuroendocrine carcinomas [35,43].

These distinctions are clinically important because SWI/SNF-deficient tumors may have different biology, prognosis and therapeutic considerations. Their recognition also reinforces a broader principle: neuroendocrine marker expression must be interpreted within the full molecular and morphologic profile of the tumor, rather than treated as a stand-alone lineage-defining feature [43].

### 10.3. Mixed Tumors, Clonal Relationships and Tumor Plasticity

Mixed neuroendocrine and non-neuroendocrine tumors provide a useful model for studying divergent differentiation. When both components share molecular alterations, this supports a common clonal origin with phenotypic divergence. This mechanism is particularly plausible in HPV-associated cervical tumors containing both neuroendocrine carcinoma and squamous or glandular carcinoma. Similar principles may apply to endometrial or ovarian tumors in which neuroendocrine and non-neuroendocrine components share driver alterations, although molecularly annotated data remain limited [13,14,74,75,76].

In other cases, neuroendocrine differentiation may reflect tumor progression, subclonal evolution or lineage plasticity. Neuroendocrine transformation is well recognized as a mechanism of progression and therapy resistance in other malignancies, particularly prostate cancer and lung cancer. In gynecologic tumors, evidence is more limited, but the concept is relevant in recurrent or treatment-resistant disease, especially when the recurrent tumor shows a morphology different from the original lesion [77,78].

Distinguishing true transformation from under-sampling of a pre-existing neuroendocrine component can be difficult. Comparison with prior pathology, repeat biopsy, expanded immunohistochemistry and molecular profiling may help clarify whether neuroendocrine differentiation represents clonal evolution, divergent differentiation or a newly recognized component of a heterogeneous tumor. This issue is clinically relevant because the emergence of a high-grade neuroendocrine phenotype may influence systemic treatment considerations [13,75,79].

### 10.4. Therapeutic Relevance and Limitations of Molecular Data

Molecular findings may influence treatment in selected settings. MMR deficiency may support immune checkpoint inhibition in advanced or recurrent gynecologic cancers. *POLE* mutation may affect prognostic assessment and, in some contexts, treatment intensity. HPV-associated biology may have implications for immune response and therapeutic strategies, although data specific to gynecologic neuroendocrine carcinoma remain limited. Actionable genomic alterations, when present, may support enrollment in molecularly guided clinical trials [1,4,33,58].

For poorly differentiated neuroendocrine carcinoma, however, treatment is still often driven by histology, stage and clinical behavior rather than by site-specific molecular algorithms. This reflects the lack of large molecularly annotated cohorts and the tendency of published series to combine pure neuroendocrine carcinomas, mixed tumors and conventional carcinomas with focal marker expression. Future studies should separate these categories to determine whether molecular subgroups have independent prognostic or predictive value [4,76].

Overall, molecular data should be used to answer specific diagnostic and clinical questions: Does the tumor fit an HPV-associated pathway? Does it belong to a recognized endometrial molecular subtype? Could it represent a SWI/SNF-deficient mimic? Are mixed components clonally related? Is there an actionable alteration? Framed in this way, molecular analysis complements morphology and immunohistochemistry and provides a bridge from diagnosis to prognosis and treatment [33,35,74,75].

## 11. Prognostic Significance

The prognostic impact of neuroendocrine differentiation in female genital tract tumors depends on what the finding actually represents. It cannot be interpreted uniformly across all tumors or sites. A true poorly differentiated neuroendocrine carcinoma, a well-differentiated ovarian neuroendocrine tumor, a mixed carcinoma with a neuroendocrine component and a conventional carcinoma with focal neuroendocrine marker expression have different clinical implications. Prognostic interpretation should therefore integrate tumor category, anatomic site, stage, morphology, molecular context and metastatic pattern [11,13,62].

### 11.1. Poorly Differentiated Neuroendocrine Carcinoma

Poorly differentiated neuroendocrine carcinoma is the category most consistently associated with adverse outcomes. These tumors are generally high-grade malignancies with rapid growth, high mitotic activity, necrosis, frequent lymphovascular invasion and early metastatic potential. This aggressive behavior is best documented in the cervix, where small-cell neuroendocrine carcinoma is associated with a higher risk of nodal and distant relapse than conventional cervical squamous cell carcinoma or adenocarcinoma [4,5,13,80].

Although data are more limited for the endometrium, ovary, vagina and vulva, poorly differentiated neuroendocrine carcinomas in these sites should also be regarded as high-risk tumors. In the endometrium, reported cases often occur in association with high-grade or mixed histology, making outcome interpretation difficult. In the ovary, prognosis depends on correct distinction from metastatic neuroendocrine carcinoma and from small-cell carcinoma of the ovary, hypercalcemic type. Vaginal and vulvar neuroendocrine carcinomas are too rare for robust risk stratification, but available reports suggest aggressive behavior [4,5,51].

Because diagnostic criteria have varied across studies, prognosis should be interpreted cautiously. Some published series may include pure neuroendocrine carcinomas, mixed tumors and conventional carcinomas with neuroendocrine marker expression under overlapping labels. This heterogeneity likely contributes to inconsistent outcome estimates and reinforces the need for standardized classification [6,13,18].

### 11.2. Well-Differentiated Neuroendocrine Tumors

Well-differentiated neuroendocrine tumors, particularly ovarian carcinoid tumors, have a different prognostic profile. When localized to the ovary and completely excised, many behave indolently. Prognosis is influenced by stage, subtype, tumor size, association with other ovarian neoplasms and completeness of resection [11,62].

The main prognostic issue in this setting is correct classification. A primary ovarian well-differentiated neuroendocrine tumor associated with a teratoma or mucinous tumor should not be grouped with poorly differentiated ovarian neuroendocrine carcinoma. Conversely, a well-differentiated neuroendocrine tumor involving the ovary may represent metastasis from a gastrointestinal or pancreatic primary, which carries different staging and management implications [4,11,62].

Outside the ovary, true primary well-differentiated neuroendocrine tumors of the female genital tract are exceptionally uncommon. Apparent well-differentiated neuroendocrine tumors in the cervix, endometrium, vagina or vulva should therefore prompt careful exclusion of metastatic disease before prognostic assumptions are made [11,13].

### 11.3. Mixed Tumors and Focal Marker Expression

The prognosis of mixed neuroendocrine and non-neuroendocrine tumors depends on the nature of both components. A poorly differentiated neuroendocrine carcinoma component may be clinically important even when it represents only part of the tumor, particularly if it is present in lymphovascular spaces, lymph nodes or distant metastases. In such cases, the neuroendocrine component may influence risk assessment and systemic treatment decisions [24,75].

However, not all mixed tumors should be interpreted in the same way. A well-differentiated neuroendocrine component in a conventional ovarian tumor has very different implications from a small-cell neuroendocrine carcinoma component in a cervical tumor. Therefore, prognostic assessment should specify the type and grade of the neuroendocrine component, its approximate extent and the component represented in metastatic sites when known [24,62].

By contrast, the prognostic significance of focal neuroendocrine marker expression in otherwise conventional gynecologic carcinoma remains uncertain. Limited expression of synaptophysin, chromogranin A, CD56 or INSM1 should not automatically be considered equivalent to neuroendocrine carcinoma. Future studies should separate such tumors from true neuroendocrine carcinomas and mixed tumors to determine whether marker expression has independent prognostic value [6].

### 11.4. Determinants and Limitations of Prognostic Assessment

Stage remains one of the most important prognostic factors across gynecologic malignancies, but tumor category modifies its interpretation. Poorly differentiated neuroendocrine carcinomas may behave aggressively even when apparently localized, especially in the cervix. Lymphovascular invasion, nodal involvement, distant metastasis, high proliferative activity and incomplete resection are adverse features. In mixed tumors, the identity of the metastatic component may provide additional prognostic information [4,80].

Molecular context may also influence prognosis, particularly in endometrial tumors. MMR deficiency, *POLE* mutation and p53-abnormal status should be considered within the established framework of endometrial carcinoma, while SWI/SNF-deficient mimics should be separated from neuroendocrine carcinoma because their biology may differ. At present, however, molecular prognostic models specific to gynecologic neuroendocrine carcinomas remain insufficiently developed [1,35].

Available prognostic data are limited by rarity, retrospective study design, small cohorts, heterogeneous treatment and inconsistent terminology. The most reliable general conclusion is that true poorly differentiated neuroendocrine carcinoma represents an aggressive phenotype, whereas well-differentiated ovarian neuroendocrine tumors and conventional carcinomas with focal marker expression should not be assumed to share the same clinical behavior. This distinction is central to therapeutic decision-making, addressed in the following section [1,13].

## 12. Therapeutic Implications

The diagnostic and prognostic distinctions discussed above have direct therapeutic relevance. Treatment should not be guided by neuroendocrine marker positivity alone but by the integrated classification of the tumor as a poorly differentiated neuroendocrine carcinoma, well-differentiated neuroendocrine tumor, mixed neoplasm, metastatic lesion or conventional carcinoma with incidental marker expression. Because gynecologic neuroendocrine neoplasms are rare, therapeutic evidence is limited and often extrapolated from small-cell lung carcinoma, extrapulmonary neuroendocrine carcinoma or site-specific gynecologic cancer management [4,24].

### 12.1. Poorly Differentiated Neuroendocrine Carcinoma

Poorly differentiated neuroendocrine carcinoma is generally managed as an aggressive systemic disease. Platinum-based chemotherapy, often combined with etoposide, is commonly used by analogy with small-cell lung carcinoma and other extrapulmonary high-grade neuroendocrine carcinomas. Surgery and radiotherapy may be incorporated depending on site, stage and resectability, but local therapy alone is often insufficient because of the high risk of systemic relapse [4,24].

Cervical neuroendocrine carcinoma is the best-established gynecologic setting in which this principle applies. Even apparently early-stage tumors may have substantial risk of nodal and distant dissemination, supporting the consideration of systemic therapy as part of multimodality treatment. In locally advanced disease, chemoradiation and systemic chemotherapy are commonly used, although optimal sequencing and regimens remain incompletely standardized [81,82].

For endometrial, ovarian, vaginal and vulvar poorly differentiated neuroendocrine carcinomas, treatment is more individualized because evidence is sparse. Surgery may be used for diagnosis, staging or cytoreduction when feasible, while chemotherapy and radiotherapy are selected according to site, stage, histologic components and patient factors. These tumors should ideally be discussed in multidisciplinary tumor boards, and clinical trial or registry enrollment should be considered whenever available [42,83].

### 12.2. Well-Differentiated Neuroendocrine Tumors and Metastatic NETs

Well-differentiated neuroendocrine tumors require a different therapeutic approach. Localized ovarian carcinoid tumors are primarily managed surgically and may have favorable outcomes when confined to the ovary and completely excised. Fertility-sparing surgery may be considered in selected patients depending on age, stage, associated components and standard gynecologic oncology principles [11,62].

Somatostatin receptor imaging and somatostatin receptor-targeted strategies are more relevant to well-differentiated neuroendocrine tumors than to poorly differentiated neuroendocrine carcinomas. Their use may be considered in selected advanced, recurrent or metastatic well-differentiated tumors when receptor expression is demonstrated. They should not be assumed to apply to high-grade neuroendocrine carcinoma without appropriate evidence [84].

If a neuroendocrine tumor involving the ovary, vagina or vulva represents metastasis from a gastrointestinal, pancreatic, pulmonary or cutaneous primary, treatment should follow the biology and stage of the primary tumor rather than gynecologic primary tumor algorithms. This reinforces the therapeutic importance of distinguishing primary gynecologic neuroendocrine neoplasms from metastatic disease [11].

### 12.3. Mixed Tumors and Conventional Carcinomas with Focal Marker Expression

In mixed neuroendocrine and non-neuroendocrine tumors, management is often influenced by the most aggressive component. A poorly differentiated neuroendocrine carcinoma component may justify consideration of neuroendocrine carcinoma-directed systemic therapy, particularly if it is substantial, high-grade or present in lymphovascular invasion, lymph nodes or distant metastases. However, treatment should also account for the associated non-neuroendocrine component, stage, molecular profile, and patient-specific factors [75,85].

Not all mixed tumors require the same approach. A well-differentiated neuroendocrine component arising in an ovarian teratoma or mucinous tumor has different implications from a small-cell neuroendocrine carcinoma component in a cervical tumor. Therefore, precise classification of each component is essential before treatment decisions are made [62].

By contrast, conventional gynecologic carcinomas with only focal neuroendocrine marker expression should generally be treated according to their established histotype, stage and molecular risk group. At present, there is insufficient evidence to justify treatment intensification solely on the basis of limited synaptophysin, chromogranin A, CD56 or INSM1 expression in the absence of supportive neuroendocrine morphology [6,62].

### 12.4. Molecularly Informed Therapy and Future Directions

Molecular findings may influence treatment in selected contexts. Mismatch repair deficiency may support immune checkpoint inhibition in advanced or recurrent gynecologic cancers. *POLE* mutation may affect prognostic interpretation and treatment intensity in endometrial carcinoma. HPV-associated biology may have immunologic relevance in cervical and some lower genital tract tumors, although data specific to neuroendocrine carcinoma remain limited. Actionable genomic alterations, when present, may support enrollment in molecularly guided clinical trials.

For poorly differentiated neuroendocrine carcinoma, however, treatment remains driven mainly by histology, stage and clinical behavior rather than by validated molecular algorithms. This reflects the rarity of these tumors and the lack of large molecularly annotated therapeutic cohorts. Future studies should distinguish true neuroendocrine carcinoma from mixed tumors and conventional carcinomas with focal marker expression, because these groups are unlikely to share the same therapeutic vulnerabilities [1,33,76,86].

Overall, the therapeutic role of neuroendocrine differentiation depends on diagnostic category. True poorly differentiated neuroendocrine carcinoma may require aggressive multimodality treatment, localized well-differentiated ovarian neuroendocrine tumors are often managed surgically, mixed tumors require component-specific interpretation, and focal marker expression alone should not drive neuroendocrine-directed therapy. This framework underscores the need for a practical diagnostic algorithm that can translate pathology findings into clinically meaningful categories [62,86].

## 13. Proposed Integrated Diagnostic—Clinical Algorithm

**Figure 4:**
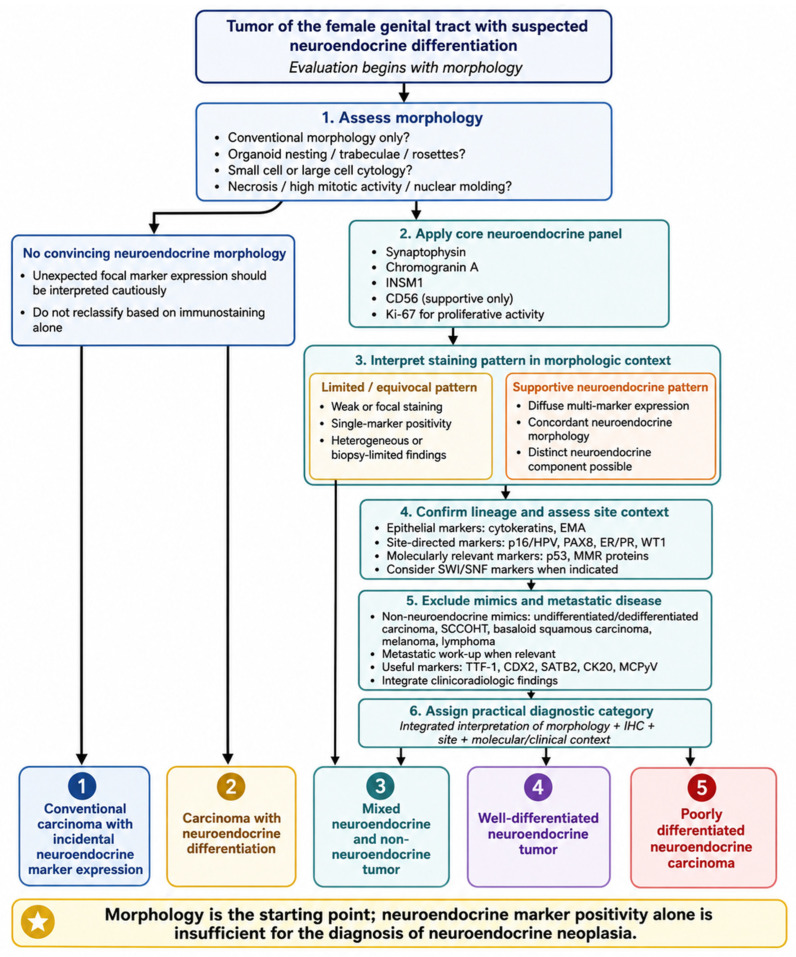
Integrated diagnostic workflow for suspected neuroendocrine differentiation in tumors of the female genital tract. The algorithm emphasizes morphology as the starting point, followed by targeted neuroendocrine immunohistochemistry, interpretation of staining patterns in morphologic context, confirmation of lineage and site, and exclusion of mimics or metastatic disease. Integrated assessment allows classification into clinically meaningful categories while avoiding reclassification based on neuroendocrine marker positivity alone.

The preceding sections support a stepwise approach to neuroendocrine differentiation in female genital tract tumors. Because neuroendocrine marker positivity alone is insufficient for diagnosis, the evaluation should begin with morphology and progress through immunohistochemical confirmation, site attribution, exclusion of mimics and final clinicopathologic categorization. The proposed algorithm is intended as a practical framework rather than a rigid rule, particularly given the rarity and heterogeneity of these tumors (Figure 4).

The first step is to determine whether the tumor shows morphologic features suggestive of neuroendocrine differentiation. These include organoid nesting, trabecular growth, rosette-like structures, peripheral palisading, nuclear molding, finely granular chromatin, necrosis, high mitotic activity or small-cell/large-cell neuroendocrine morphology. In tumors with entirely conventional morphology, unexpected focal neuroendocrine marker expression should be interpreted cautiously and should not prompt automatic diagnostic reclassification [6,13].

If morphology raises suspicion, a core neuroendocrine panel should be applied. Synaptophysin, chromogranin A and INSM1 represent a useful combination in routine practice, while CD56 may be added as a supportive but nonspecific marker. Ki-67 may help characterize proliferative activity, particularly when distinguishing a well-differentiated neuroendocrine tumor from a poorly differentiated neuroendocrine carcinoma in the appropriate context. The diagnostic value of these markers depends on whether their expression is concordant with the morphologic impression [10].

The next step is to assess the pattern of staining. Diffuse expression of multiple neuroendocrine markers in areas with neuroendocrine architecture supports clinically meaningful neuroendocrine differentiation. By contrast, weak, focal or single-marker positivity in a morphologically conventional carcinoma is insufficient for reclassification as neuroendocrine carcinoma. When possible, the report should describe whether staining is focal, patchy or diffuse, whether it is weak or strong, and whether it corresponds to a morphologically distinct component [6,13].

After neuroendocrine differentiation is suspected or confirmed, epithelial lineage and site context should be established. Cytokeratins and EMA help confirm carcinoma, while p16/HPV testing, PAX8, ER/PR, WT1, p53, MMR proteins, CK7/CK20 and other site-directed markers should be selected according to anatomic location and differential diagnosis. Molecularly relevant markers, including SWI/SNF components, may be required when undifferentiated carcinoma, dedifferentiated carcinoma or small-cell carcinoma of the ovary, hypercalcemic type, is considered [6,35].

Metastatic disease and non-neuroendocrine mimics should then be excluded. Clinical history, imaging findings and selected immunohistochemical markers such as TTF-1, CDX2, SATB2, CK20 and Merkel cell polyomavirus may be useful, but none should be interpreted in isolation. If the primary site remains uncertain, the report should state the differential diagnosis and recommend clinicoradiologic correlation rather than assigning an unsupported origin [29].

After the integration of morphology, immunohistochemistry, site context and clinical findings, most tumors can be assigned to one of five practical categories. Conventional carcinoma with incidental neuroendocrine marker expression should be diagnosed when the tumor retains conventional morphology and shows only focal, weak or scattered marker positivity. Carcinoma with neuroendocrine differentiation may be appropriate when neuroendocrine morphology and immunophenotype are present but incomplete, focal or insufficient for definitive classification as neuroendocrine carcinoma. Mixed neuroendocrine and non-neuroendocrine tumor should be diagnosed when distinct morphologic components are present. Well-differentiated neuroendocrine tumor should be reserved for tumors with well-differentiated neuroendocrine morphology and immunophenotype, most commonly in the ovary, after metastatic disease has been considered. Poorly differentiated neuroendocrine carcinoma should be used for high-grade tumors with small-cell or large-cell neuroendocrine morphology and supportive immunophenotype, after the exclusion of relevant mimics and metastases [6,13,29].

The final pathology report should translate this categorization into clinically useful information. It should specify the presence and extent of neuroendocrine morphology, markers performed, staining pattern, proliferation index if assessed, associated non-neuroendocrine component, lymphovascular invasion, metastatic component and relevant molecular findings. In ambiguous cases, diagnostic uncertainty should be stated explicitly. For example, a limited biopsy may be best reported as “high-grade carcinoma with neuroendocrine differentiation” rather than definitive neuroendocrine carcinoma if architecture is insufficient. Similarly, a conventional carcinoma with focal marker expression may include a comment stating that the findings do not support neuroendocrine carcinoma [1,7,59].

For mixed tumors, the report should indicate whether the neuroendocrine component is poorly differentiated and whether it is present in lymphovascular spaces, lymph nodes or distant metastases. For ovarian tumors, the report should comment on features supporting primary origin versus metastasis when relevant. For endometrial tumors, molecular findings should be integrated into the interpretation when available [1,56].

This algorithm emphasizes that neuroendocrine marker expression is only the starting point of evaluation, not the endpoint. Its purpose is to reduce overdiagnosis, avoid the underrecognition of aggressive neuroendocrine carcinoma and translate complex pathology findings into diagnostic categories that are meaningful for prognosis and treatment [87].

## 14. Conclusions

Neuroendocrine differentiation in tumors of the female genital tract is a heterogeneous clinicopathologic phenomenon with variable diagnostic, prognostic, and therapeutic significance. Its interpretation requires the separation of incidental neuroendocrine marker expression from carcinoma with neuroendocrine differentiation, mixed neuroendocrine/non-neuroendocrine tumors, well-differentiated neuroendocrine tumors, and poorly differentiated neuroendocrine carcinomas.

Morphology remains the starting point for diagnosis, while immunohistochemistry should be used as a supportive and contextual tool. Core neuroendocrine markers, including synaptophysin, chromogranin A, CD56, and INSM1, should be interpreted according to staining pattern, extent, and concordance with architectural and cytologic features. Ancillary non-neuroendocrine markers are essential for confirming epithelial lineage, assigning tumor origin, identifying molecularly defined mimics, and excluding metastatic disease.

A site-specific, panel-based approach may help avoid both overdiagnosis of neuroendocrine carcinoma in tumors with limited marker expression and underrecognition of clinically meaningful neuroendocrine differentiation. Future multi-institutional and molecularly annotated studies are needed to refine diagnostic criteria, clarify prognosis, and determine whether neuroendocrine differentiation has independent biological or therapeutic relevance in gynecologic malignancies.

## Figures and Tables

**Table 1:** Immunohistochemical assessment of neuroendocrine differentiation in tumors of the female genital tract: core neuroendocrine markers and ancillary non-neuroendocrine markers.

Marker		Typical Staining Pattern	Main Diagnostic Value	Key Limitations/Pitfalls	Practical Interpretation
Core neuroendocrine markers	Synaptophysin	Cytoplasmic, often diffuse in true neuroendocrine tumors and carcinomas	Sensitive marker of neuroendocrine differentiation; useful as part of the initial panel	Not entirely specific; focal or patchy expression may occur in conventional gynecologic carcinomas	Strongest when diffuse and concordant with neuroendocrine morphology
	Chromogranin A	Cytoplasmic granular staining	More specific marker; particularly useful in well-differentiated neuroendocrine tumors	Less sensitive in poorly differentiated neuroendocrine carcinomas; may be weak or negative	Negative staining does not exclude neuroendocrine carcinoma if morphology and other markers support the diagnosis
	CD56	Membranous and/or cytoplasmic staining	Sensitive supportive marker	Low specificity; isolated positivity is a common diagnostic pitfall	Should not be used alone to diagnose neuroendocrine differentiation or neuroendocrine carcinoma
	INSM1	Nuclear staining	Useful marker, especially in poorly differentiated neuroendocrine carcinoma; easier to interpret in crushed or small biopsy material	Not completely specific; must still be interpreted in morphologic context	Strong nuclear staining supports neuroendocrine differentiation when morphology is appropriate
Ancillary non-neuroendocrine markers	Ki-67	Nuclear proliferation index	Helps assess proliferative activity and supports distinction between well-differentiated NET and poorly differentiated NEC in the appropriate context	Does not establish neuroendocrine lineage	Should be interpreted as a proliferation marker, not as evidence of neuroendocrine differentiation
	Cytokeratins/EMA	Cytoplasmic and/or membranous epithelial staining	Confirm epithelial lineage in poorly differentiated tumors	May be reduced or focal in some high-grade tumors	Essential before diagnosing poorly differentiated neuroendocrine carcinoma, especially in small round-cell tumors
	p16/HPV testing	p16 block-type staining; HPV detected by ISH or molecular methods	Supports HPV-associated cervical or lower genital tract origin when morphology and clinical context fit	p16 is not site-specific and can be positive in non-cervical high-grade tumors	Most useful in cervical tumors and selected vaginal/vulvar tumors
	PAX8, ER, PR, WT1	Nuclear staining, depending on marker	Supports Müllerian, endometrial or ovarian lineage in appropriate settings	None is definitive alone; expression may vary by tumor type and differentiation	Used as site-directed markers within a broader panel
	p53/MMR proteins	p53 wild-type or abnormal pattern; retained or lost MMR protein expression	Provides molecular context, especially in endometrial tumors	Does not define neuroendocrine differentiation	Useful for classifying high-grade endometrial tumors and identifying therapeutically relevant subgroups
	SMARCA4/SMARCB1/SWI/SNF markers	Retained or lost nuclear expression	Helps identify SWI/SNF-deficient mimics such as dedifferentiated carcinoma or SCCOHT	Should be applied selectively based on morphology and clinical context	Loss of expression may redirect diagnosis away from neuroendocrine carcinoma
	TTF-1, CDX2, SATB2, CK20, MCPyV	Site-dependent nuclear, cytoplasmic or membranous patterns	Helps evaluate metastatic pulmonary, gastrointestinal, pancreatic or Merkel cell origin	Expression overlap may occur; no marker proves origin in isolation	Must be interpreted with clinical history, imaging and disease distribution

**Table 2:** Practical diagnostic wording and reporting recommendations for tumors with neuroendocrine marker expression or neuroendocrine differentiation.

Scenario	Findings	Preferred Diagnostic Wording	Suggested Comment	Diagnostic Rationale
Conventional carcinoma with focal neuroendocrine marker expression	Conventional morphology; focal or weak staining for one or more neuroendocrine markers; no neuroendocrine architecture or cytology	“Conventional carcinoma, with focal neuroendocrine marker expression”	“The findings do not support a diagnosis of neuroendocrine carcinoma.”	Avoids overdiagnosis and inappropriate treatment intensification
Isolated CD56 positivity	Conventional morphology; CD56-positive; synaptophysin, chromogranin A and/or INSM1 negative or non-supportive	Do not diagnose neuroendocrine differentiation based on CD56 alone	“CD56 expression is nonspecific and, in isolation, is insufficient for neuroendocrine classification.”	Prevents misclassification based on a low-specificity marker
Partial morphology and partial IHC support	Focal organoid/trabecular growth, rosettes or nuclear molding; limited or heterogeneous neuroendocrine marker expression	“Carcinoma with neuroendocrine differentiation”	“The neuroendocrine features are focal/limited and insufficient for definitive classification as neuroendocrine carcinoma.”	Communicates uncertainty while acknowledging a potentially relevant phenotype
Limited biopsy with high-grade neuroendocrine features	Small biopsy shows high-grade carcinoma with neuroendocrine morphology and supportive markers, but tumor extent cannot be assessed	“High-grade carcinoma with neuroendocrine differentiation” or “Poorly differentiated neuroendocrine carcinoma, if supported by morphology and diffuse markers”	“Definitive classification and assessment of any associated non-neuroendocrine component may require evaluation of the resection specimen.”	Avoids overquantification and recognizes sampling limitations
Distinct neuroendocrine and non-neuroendocrine components	Separate morphologic components, each supported by appropriate IHC	“Mixed neuroendocrine and non-neuroendocrine tumor”	“Both components should be reported, with approximate proportions when possible.”	Ensures that the clinically aggressive component is not missed
Poorly differentiated neuroendocrine carcinoma	Classic small-cell or large-cell morphology; necrosis; high mitotic activity; diffuse multimarker neuroendocrine expression	“Poorly differentiated neuroendocrine carcinoma, small-cell type” or “large-cell type”	“Correlation with site-specific findings and exclusion of metastatic disease are recommended when clinically indicated.”	Identifies a high-risk tumor category with therapeutic implications
Well-differentiated ovarian neuroendocrine tumor	Well-differentiated neuroendocrine morphology; supportive markers; ovarian setting, often with teratomatous or mucinous elements	“Well-differentiated neuroendocrine tumor of the ovary”	“Features supporting primary ovarian origin versus metastasis should be documented.”	Separates indolent primary ovarian NETs from aggressive NECs and metastases
Possible metastatic neuroendocrine neoplasm	Neuroendocrine morphology and markers; atypical site, bilateral ovarian disease, extraovarian disease or clinical history of extragenital NET/NEC	“Neuroendocrine neoplasm involving [site], favor metastatic origin” or “primary site uncertain”	“Clinicoradiologic correlation is required to determine the primary site.”	Prevents unsupported assignment of a gynecologic primary

## Data Availability

No new data were created or analyzed in this study. Data sharing is not applicable to this article.
